# Prevalence of Serious Bacterial Infections Among Febrile Infants 90 Days or Younger in a Canadian Urban Pediatric Emergency Department During the COVID-19 Pandemic

**DOI:** 10.1001/jamanetworkopen.2021.16919

**Published:** 2021-07-13

**Authors:** Brett Burstein, Gregory Anderson, Alexandra Yannopoulos

**Affiliations:** 1 Montreal Children’s Hospital, Division of Pediatric Emergency Medicine, McGill University, Montreal, Quebec, Canada; 2 Department of Biostatistics, Epidemiology and Occupational Health, McGill University, Montreal, Quebec, Canada; 3 Research Institute of the McGill University Health Centre, Montreal, Quebec, Canada

## Abstract

This cross-sectional study compares the prevalence of severe bacterial infections in febrile neonates and infants before vs during the COVID-19 pandemic in Montreal, Quebec, Canada.

## Introduction

Approximately 2% of all full-term neonates are evaluated for fever in the first months of life, and there exists significant ongoing variation in their care.^1^ Although most febrile neonates and infants 90 days or younger (hereafter referred to as *young infants*) have self-limited viral illnesses, the prevalence of life-threatening serious bacterial infections (SBIs) has remained approximately 10% for more than 30 years.^1^ On March 11, 2020, the COVID-19 pandemic was declared, and since that time, studies worldwide have reported a 30% to 89% reduction in the number of children brought to emergency care^2,3^ as well as fewer circulating respiratory viruses owing to widespread COVID-19 public health mitigation strategies.^4,5^ To our knowledge, the epidemiologic characteristics of SBIs among febrile young infants during the COVID-19 pandemic has not previously been assessed. We sought to assess the prevalence of SBIs among febrile young infants evaluated in the emergency department (ED) for fever during the COVID-19 pandemic.

## Methods

We undertook a retrospective cross-sectional study of prospectively collected data from a quality improvement database for young infants who were evaluated for fever from March 18, 2018, to March 17, 2021, at an urban tertiary pediatric ED in Montreal, Quebec, the COVID-19 first-wave epicenter in Canada. Standardized clinical, laboratory, and follow-up data were collected for all young infants presenting to the ED with a history of or current rectal temperature of 38.0 °C or greater. Diagnostic testing was preformed at the discretion of the treating physician. All young infants who did not have blood or cerebrospinal fluid cultures performed were followed up by telephone to ascertain for missed SBIs. On March 18, 2020, universal targeted testing for SARS-CoV-2 replaced multiplex respiratory viral testing (13-virus panel) for febrile young infants. Severe bacterial infections were defined as urinary tract infections, bacteremia, or bacterial meningitis; bacteremia and bacterial meningitis were considered invasive bacterial infections (IBIs).^1^ The primary outcomes were the proportions of SBIs and IBIs among all young infants evaluated for fever. Outcomes were compared during and before the pandemic using a χ^2^ test with Stata, version 14.1 (StataCorp LLC), and a 2-tailed *P* < .05 was considered statistically significant. This study received approval from the McGill University Health Centre Research Ethics Board, with a waiver of informed consent because data were collected prospectively for quality improvement purposes. The study followed the Strengthening the Reporting of Observational Studies in Epidemiology (STROBE) reporting guideline.

## Results

During the COVID-19 pandemic period (March 2020 to March 2021), 324 young infants (202 male [62.3%]; median age, 53 days [interquartile range (IQR), 32-67 days]) were evaluated for fever compared with 951 from March 2018 to March 2019 (543 male [57.1%]; median age, 55 days [IQR, 38-73 days]) and 973 from March 2019 to March 2020 (581 male [59.7%]; median age, 54 days [IQR, 38-71 days]) (Table). Demographic characteristics, diagnostic testing, and ED disposition of the young infants evaluated did not change throughout the study period. Multiplex virus testing was performed for 1218 of 1924 young infants (63.3%) before the pandemic, and 311 of 324 young infants (96.0%) underwent SARS-CoV-2 testing during the pandemic.

**Table.** Demographic Characteristics, Diagnostic Testing, and ED Disposition of Febrile Neonates and Infants 90 Days or Younger Before and During the COVID-19 Pandemic^a^

Characteristic	Prepandemic cohort		Pandemic cohort: March 2020-March 2021 (n = 324)
	March 2018-March 2019 (n = 951)	March 2019-March 2020 (n = 973)	
Age, d			
Median (IQR)	55 (38-73)	54 (38-71)	53 (32-67)
0-30	169 (17.8)	173 (17.8)	78 (24.1)
31-60	374 (39.3)	407 (41.8)	123 (38.0)
61-90	408 (42.9)	393 (40.4)	123 (38.0)
Sex			
Female	408 (42.9)	392 (40.3)	122 (37.7)
Male	543 (57.1)	581 (59.7)	202 (62.3)
Triage acuity level			
Immediate	4 (0.4)	7 (0.7)	1 (0.3)
Emergent	681 (71.6)	676 (69.5)	256 (79.0)
Urgent	252 (26.5)	279 (28.7)	62 (19.1)
Semi-urgent	13 (1.4)	10 (1.0)	4 (1.2)
Nonurgent	1 (0.1)	1 (0.1)	1 (0.3)
Disposition			
Discharged from ED	645 (67.8)	700 (71.9)	209 (64.5)
Hospitalized	306 (32.2)	273 (28.1)	115 (35.5)
Total hospital length of stay, median (IQR), h	6.8 (4.1-41.3)	6.4 (3.8-34.9)	6.5 (4.3-50.7)
Diagnostic testing			
Blood culture			
Total tests	704 (74.0)	676 (69.5)	240 (74.1)
Positive results	13 (1.8)	8 (1.2)	11 (4.6)
Urine culture			
Total tests	756 (79.5)	748 (76.9)	265 (81.8)
Positive results	97 (12.8)	86 (11.5)	63 (23.8)
CSF culture			
Total tests	190 (20.0)	211 (21.7)	101 (31.2)
Positive results	4 (2.1)	2 (1.0)	2 (2.0)
Multiplex viral testing			
Total tests	633 (66.6)	585 (60.1)	25 (7.7)
Positive results	409 (64.6)	365 (62.4)	9 (36.0)
SARS-CoV-2			
Total tests	0	2 (0.2)	311 (96.0)
Positive results	NA	0	20 (6.4)
Bacterial infections			
SBI	102 (10.7)	90 (9.2)	66 (20.4)
IBI	14 (1.5)	8 (0.8)	11 (3.4)
Coinfections, No/total No. (%)			
SBI with positive multiplex viral test result	31/409 (7.6)	23/365 (6.3)	2/9 (22.2)
SBI with positive SARS-CoV-2 test result	NA	0/2 (0)	1/20 (5.0)
IBI with positive multiplex viral test result	1/409 (0.2)	1/365 (0.3)	0/9 (0)
IBI with positive SARS-CoV-2 test result	NA	0/2 (0)	0/20 (0)
Overall ED visits, No.			
Patients aged <18 y	77 157	77 182	39 845
Patients aged <90 d	3768	3522	2044

Abbreviations: CSF, cerebrospinal fluid; ED, emergency department; IBI, invasive bacterial infection; IQR, interquartile range; NA, not applicable; SBI, serious bacterial infection.

^a^ Data are presented as number (percentage) or patients unless otherwise indicated.

During the pandemic, the absolute number of young infants presenting with fever decreased 66.3%; however, the proportion of those with SBI increased significantly (192 of 1924 [10.0%] before the pandemic vs 66 of 324 [20.4%] during the pandemic; *P* < .001) (Table and Figure). The absolute number of IBIs remained stable, but the proportion increased by 3 fold (22 of 1924 [1.1%] before the pandemic vs 11 of 324 [3.4%] during the pandemic; *P* = .005). Of 311 young infants tested for SARS-CoV-2, 20 had positive results; all had mild symptoms, and 4 (all younger than 28 days) were hospitalized (mean length of stay, 48.9 hours [range, 32.3-61.0 hours]). One young infant discharged from the ED with SARS-CoV-2 infection (5.0%) had a concomitant urinary tract infection; this prevalence of coinfection was similar to that among young infants with SBI before the pandemic (54 of 774 [7.0%]) (*P* > .99).

**Figure.**
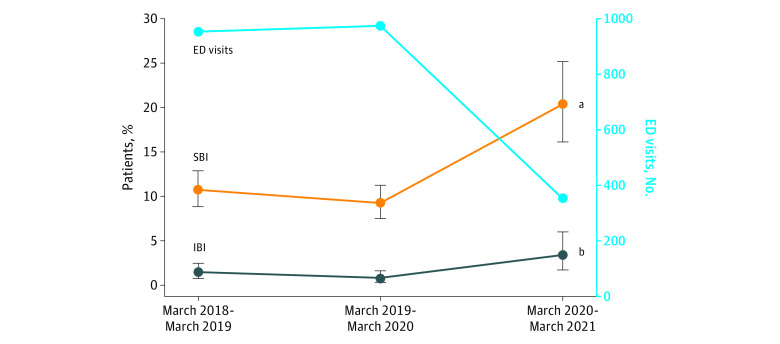
Infections and Emergency Department (ED) Visits Among Febrile Neonates and Infants 90 Days or Younger Before and During the COVID-19 Pandemic IBI indicates invasive bacterial infection; SBI, severe bacterial infection. ^a^*P* < .001 for the proportion of patients with SBI during vs before the COVID-19 pandemic. ^b^*P* = .005 for the proportion of patients with IBIs before vs during the COVID-19 pandemic.

## Discussion

During the COVID-19 pandemic, ED visits among febrile young infants decreased 66.3%; however, among those brought to the ED, the prevalence of SBIs doubled and the prevalence of IBIs tripled. To our knowledge, this is the largest analysis of febrile young infants during the COVID-19 pandemic and the first to identify an increase in SBI prevalence during this period. These findings suggest that a high degree of caution is necessary when evaluating febrile young infants while COVID-19 public health strategies continue to be implemented.

Overall, the number of SARS-CoV-2 infections among febrile young infants was low compared with that in a small cohort of neonates and infants younger than 57 days in a study from March to April 2020, in which 20 of 30 patients tested positive of SARS-CoV-2.^6^ Neonates and infants with COVID-19 in that cohort had mild symptoms.^6^ In addition, the present analysis found that the proportion of concomitant SBIs among young infants with SARS-CoV-2 infection was similar to the proportion of bacterial coinfection with other respiratory viruses before the pandemic.

A limitation of this study is that tests were infrequently performed for respiratory viruses other than SARS-CoV-2 during the pandemic because reagents were in critical shortage. Also, this was a single-center study at an urban tertiary pediatric ED in a city with a high prevalence of COVID-19.
